# "The evil virus cell": Students‘ knowledge and beliefs about viruses

**DOI:** 10.1371/journal.pone.0174402

**Published:** 2017-03-28

**Authors:** Uwe K. Simon, Sonja M. Enzinger, Andreas Fink

**Affiliations:** 1 Center for Didactics of Biology, Karl-Franzens-University Graz, Schubertstraße, Graz, Austria; 2 Department of Biology, University of Teacher Education Weingarten, Kirchplatz, Weingarten, Germany; 3 Institute of Psychology, Karl-Franzens-University Graz, Universitätsplatz 2/DG, Graz, Austria; University of St Andrews, UNITED KINGDOM

## Abstract

Education about virus biology at school is of pivotal interest to raise public awareness concerning means of disease transmission and, thus, methods to prevent infection, and to reduce unnecessary antibiotic treatment due to patient pressure on physicians in case of viral diseases such as influenza. This study aimed at making visible the knowledge of Austrian high school and university students with respect to virus biology, virus structure and health-education issues. The data presented here stem from comprehensive questionnaire analyses, including the task to draw a virus, from a cross-sectional study with 133 grade 7 and 199 grade 10 high school students, and 133 first-year biology and 181 first-year non-biology university students. Analyses were performed both quantitatively and qualitatively. ANOVA revealed a highly significant group effect for total knowledge relating to virus biology and health issues (*F*(3, 642) = 44.17, *p* < 0.01, η^2^p = 0.17). Specific post-hoc tests by means of the Tukey test showed significant differences between all groups (*p* < .01) with the exception of 1^st^ year non-biology students and grade 10 high school students. Students enrolled in university-level biology outperformed all other groups, even though they had not yet encountered this topic at their courses; part of this phenomenon might be due to their affinity for learning about biological topics. However, even many first-year biology students had a high number of severe misconceptions, e.g., defining a virus as a pro- or eukaryotic cell, or falsely naming malaria as a viral disease. Since there was no significant difference in virus-related knowledge between high schools, virus biology seems to have been taught similarly among the tested schools. However, the majority of participants stated that the virus-related knowledge they had acquired at school was not sufficient. Based on the results presented here we urgently suggest improving and intensifying teaching this topic at school, since virus-related knowledge was by far too fragmentary among many participants. Such lack of health-relevant knowledge may contribute to pressure on physicians by patients to unnecessarily prescribe antibiotics, and possibly lead to potentially dangerous neglect concerning vaccination. The effectiveness of newly developed virus-related teaching units and material could be tested with the instrument used here.

## Introduction

Hardly any year passes by without a viral disease catching public attention. Recently, the Zika virus was on the run, both as an epidemic and in the media. Viruses influence our lives in many respects. Most often viruses are viewed negatively, since we encounter them mainly in the form of diseases. The current study was sparked by the intensive media coverage about Ebola in 2014 and curiosity about what students of various age groups knew (or thought they know) about viruses. Viruses as a topic in the media may influence students’ perception of the importance of virus-related knowledge as well as their own factual knowledge. This may not always happen directly, especially with younger students, but may be mediated by other family members. In 2014, the year our questionnaire was distributed, the ORF (Austrian state television) had viruses as a topic in 201 TV-programs. The four most widely read Austrian newspapers (Presse, Standard, Kleine Zeitung, Kurier) reported 981 times in total about virus-related topics, spanning from 83 (Presse) to 396 (Standard) with Ebola as by far the most frequently discussed disease. Articles referring to charity events, movies, computer software, etc., were not included in this count. Naturally, the danger of transmission and how to prevent it was central in most newspaper texts.

Educational studies about virus-related issues have hitherto also mostly been interested in knowledge about ways of transmission and prevention of infection. Only a very limited number of studies analyzed students’ biological knowledge about viruses (e.g. virus structure as distinct from pro-/eukaryotes, the cellular infection process, details about the immune system response), and, if so, usually focused on single or few aspects only [1–3]. As far as we know, very few studies have analyzed the relevant knowledge in depth and tried to improve understanding concerning virus biology, with, however, a very limited number of participants (N = 10–54) [4–6]. Here, we attempted to make visible such knowledge in a much broader way, both within and between three age groups of students (high school grade 7, high school grade 10, biology and non-biology first-year university students). Due to the large number of participants we were able to both derive quantitative measures and qualitative data from distributed questionnaires, which comprised mostly open-response items.

A second reason for this study was cumulative evidence that many clinicians and general practitioners sometimes unnecessarily prescribe antibiotics, e.g. for patients with acute respiratory infections [7–9]. Most often, this is not due to lack of relevant knowledge. Instead, psychological factors such as tiredness and patients’ (or parents’) insistence on treatment with antibiotics become important [7,9]. Consequently, some authors recently postulated a behavioral training for physicians[8,9]. But what about viral education at school? As Keselman et al. (2007) stated health “has received relatively little attention in the science education research community” (p. 845). However, Dumais and Hasni (2009) noted that “understanding the conceptions that young people have about viruses … may be important for the development of effective school health education programs” (p. 62). Furthermore, certain biological knowledge may be a prerequisite to effectively evaluate information resulting in informed decision making concerning health issues [10]. If patients knew that viruses have, for example, no cell wall and thus antibiotics interfering with cell wall synthesis are inefficient in treating viral infections, pressure on physicians for prescribing such medication might decrease.

In this study, we analyzed what students of various age groups know about viruses and how interested they are in this topic. Our hypothesis was that knowledge would increase as a function of age, though university students studying biology might be better informed about viruses than their peers studying different subjects due to possibly higher interest in viruses.

## Methods and methods

### Participants

Participants comprised 136 grade 7 and 207 grade 10 students from five Styrian high schools (Austria), along with 133 first-year biology students and 184 first-year non-biology students (mainly languages and/or history) from the University of Graz (Austria). The study took place in November 2014. These study groups were chosen for two reasons: First, viruses and viral diseases are topics in the Austrian curriculum in grades 6, 8 and 9 and may also be discussed in grade 12, but may have been taught at different times during the school year. Students from the following years were thus assumed to have incorporated comparable content at school and, with the long summer break in between, to have retained comparable knowledge. On the other hand, university students in their first semester were chosen, because they had not yet encountered virology in their courses, even if they studied biology. The underlying assumption of comparing biology students with those from the languages and arts departments was that the first might have acquired and retained more biology-related knowledge because of the presumed larger interest in biological topics.

Permission for participation and ethical approval of all procedures were given by the federal states’ school authority (Landesschulrat Steiermark), which is the institution responsible for studies involving high school students. Parents, schoolmasters, and teachers were additionally informed prior to study begin by an information sheet. The information sheet for participants' parents was distributed by the biology teacher of each class. Parents were asked to notify the teacher or researcher, if they did not want their child to take part in the study, which happened in one case. This child did not fill out the questionnaire. Students worked on the questionnaires during regular biology lessons.

At university, first-year students were introduced to the project by the first author directly prior to questionnaire administering during a regular biology and a regular German lecture, respectively. The author was new to all students and not involved in these courses to avoid influencing students’ answering due to possibly perceived dependencies. Students were told that participation in this study was voluntary. To the author’s knowledge, all students present during these lectures returned the questionnaires. However, three students did not provide their study program and were thus excluded from further analyses. Two students in the German course were also studying biology and were thus transferred to the biology group.

All participants were informed that the data acquired would be used for refining school and possibly university teaching, and that honest answering would thus be essential in their own interest and that of future student generations.

Since questionnaires were filled out using codes only comprehensible for the individual student, anonymity was granted. These codes were used for data handling, and no further contact between researcher and participants followed.

Participants had 45 min to complete the questionnaire; most returned it after approximately 20 min.

### Questionnaire

The vast majority of studies concerning concepts about viruses are based on multiple-choice or even simpler yes/no/don’t know options. This, however, can only give a very limited insight into what participants know and believe [11]. As a consequence, the questionnaire used in this study (developed by UKS) comprised mostly constructed response items, because it was the intention to make visible actively accessible knowledge and thus knowledge which would more likely be used for decision making than knowledge which required a stimulus to become visible. Constructed response items should furthermore reduce the possibility that students are misled by phrasing, e.g. by confusing ‘antibiotics’ and ‘antibodies’ [12]. Third, it was the intention to increase the variety of answers compared to multiple-choice items. Consequently, a broad spectrum of topics was covered including structure of a virus, its definition, its proliferation in the human body, its spread across hosts and prevention of infection, vaccination, host spectrum, and own experience with viral diseases. Additionally, several questions related to the Ebola epidemic, which was extensively covered in the media during the time of questionnaire administration (S1 Questionnaire).

The questionnaire was piloted with two grade 7 (N = 35) and two grade 10 biology classes (N = 37) from one high school different to those used in the main study. Additionally, a biology teacher student filled out the questionnaire on a voluntary basis and each item was subsequently discussed between student and first author. Based on these pilot data, which were not used for final analyses, the questionnaire was slightly refined. For example, the item “Describe, how a virus proliferates” was extended to “Describe, how a virus proliferates in the human body”, because a few students had thought that the means of transmission (e.g. droplet infection) were asked for instead of the replication process in the host cell. However, in general answers indicated that the items were well understood.

In the final study, questionnaires from three grade 7 students, eight grade 10 students, and three non-biology first year university students had to be discarded because of either non-serious answering (several high school students) or too much missing data. Thus, the following number of questionnaires was finally used for analyses (Table 1):

**Table 1:** Number, sex and age of participants in each study group.

Group	Total	Females	Males	No entry	Mean age	No entry	Age range
7^th^ grade high school	133	77	56	-	12.33	-	11–14
10^th^ gradehigh school	199	102	95	2	15.43	1	15–18
1^st^ year biology university	133	109	24	-	20.28	-	17–37
1^st^ year non-biology university	181	148	33	-	20.44	-	17–59

If applicable, questionnaire data from open response items were translated into pre-categories. For example, notes showing that the student differentiated between a virus and a bacterium were translated as “viruses ≠ bacteria”, while names of diseases were left as such. For health education related items (e.g. modes of infection, prevention) very many students mentioned “bodily fluids” for means of transmission, but some others referred to “saliva”, “blood”, etc. These answers were all subsumed in the pre-category “bodily fluids.” Pre-categories were further categorized into thematic groups by UKS, and mean values for thematic groups (number of concepts divided by number of students in this group) were calculated to account for different group sizes (S1–S12 Tables).

Answers for items used to assess knowledge of virus biology and related health issues were rated according to pre-defined knowledge levels (S12 Table), while drawings were rated according to categories based upon similarity with specific virus types and their correctness (S13 Table), or, if no similarity to a specific virus structure were recognizable, according to their graphical appearance (e.g. “circle”) by UKS. To reduce bias, all answers relating to general knowledge about viruses (excluding Ebola-specific and multiple-choice items) were additionally rated by SME (a biology teacher). Similarly, 20% of all drawings were coded and rated by SME. All data and ratings for all participants can be found in S14 Table.

Inter-rater reliability was calculated using a two-way mixed, absolute, intra-class correlation (ICC) [13]. ICC-values can be found at the lower end of each excel-sheet in S14 Table for each constructed response item and drawings. Since ICC-values were in the range of 0.63/0.77 (single/average measure) and 1.0 with most ICCs close to 1.0, inter-rater reliability was deemed sufficient. Differences between raters occurred almost exclusively for drawings and especially for drawings from high school students.

The overall virus knowledge was calculated as the sum of knowledge levels gained for all items relevant for general virus biology and virus health education, starting with “1” as the lowest knowledge level for each item, which indicates no or an incorrect or unspecific answer. Since 11 items with various levels were analyzed for this part of the study, the overall knowledge score ranged from 11 (minimum, and thus overall baseline) to 46 (maximally achievable knowledge levels as the sum from all items) (S12 Table). These 36 levels were additionally split into four knowledge classes to reduce the influence of chance answering, with class 1 ranging from 11 to 19, class 2 from 20 to 28, class 3 from 29 to 37, and class 4 from 38 to 46. These knowledge levels/classes do not reflect "real" (absolute) knowledge levels, but relative levels. This means that the same levels may be achieved by different answers (e.g. by providing different, but correct keywords).

The different number of levels maximally achievable (score spread) varies between items, because the items ask for different levels of complexity and depth of knowledge: Fully and correctly answering all items requiring knowledge only taught in higher levels at school (or not even than) will result in higher levels than more or less "common knowledge" (e.g. how to prevent becoming infected). This relates to all items concerning specific virology knowledge: structure (drawing), description, multiplication, and the immune system's response to a viral infection; additionally, it relates to potential hosts for viruses, since only few students might know that even fungi can be infected by viruses, while plants (tobacco mosaic virus) and bacteria (bacteriophages) are occasionally named as hosts at school. Thus, the more specific knowledge a student would need to fully understand a specific topic, the higher the knowledge levels she/he could achieve for an item.

Concepts from brainstorming (first item in questionnaire) were regarded for other items if applicable and unambiguous. For example, if a student wrote “Ebola” and “malaria” during the brainstorming, but did not mention these diseases at the item asking for viral diseases, “Ebola” would have been added as a correct viral disease there, while “malaria” would have been added as a wrong example. Similarly, if a student showed during brainstorming, that she/he understood a virus as something different from bacteria, this would have been positively rated for the item “virus description.” Such transferred concepts are shown in italics in S14 Table.

For additional coding rules and a more detailed explanation of the rating system see S12 Table.

### Statistical analyses

Spearman’s rho was used to assess associations among virus-related knowledge and demographic variables. Knowledge-related differences between the four groups (biology university students, non-biology university students, grade 7 and grade 10 high school students) were tested by means of analyses of variance (ANOVA); specific post hoc tests were performed with the Tukey Honestly Significant Difference (HSD) test. Estimates of effect sizes are given in terms of partial eta-squared measures (ηp^2^). All statistical tests were performed with α = .05 (two-tailed). Missing data were excluded from analyses.

## Results

### Demographic data

Age of participants ranged from 11 to 59 years, with an average age of 12 for grade 7, 15 for grade 10, and 20 for university students. One student did not provide any age information. 67.5% of all participants were female, 32.2% male. Two students did not provide a sex. Concerning mother tongue, 85.3% named Austrian/German, 4.6% referred to languages spoken in the Balkan states, 1.7% were northern or western European, 2.2% east European, 0.6% south European, and 5.3% non-European (e.g. Chinese, Arabian, African). Two students did not provide information concerning mother tongue. With respect to family members working in biological or medical jobs, 50.3% did not fill in any number. Those who did had mostly one or two family members working in such fields (22.6% and 13.8%, respectively) with a few giving higher numbers. Concerning university students, 58.6% had been to school in Styria (the Austrian state this study took place in), 33.8% came from other Austrian states, and 7.3% were foreigners. One student did not mention any state.

### Overall virus-related knowledge

**Fig 1:**
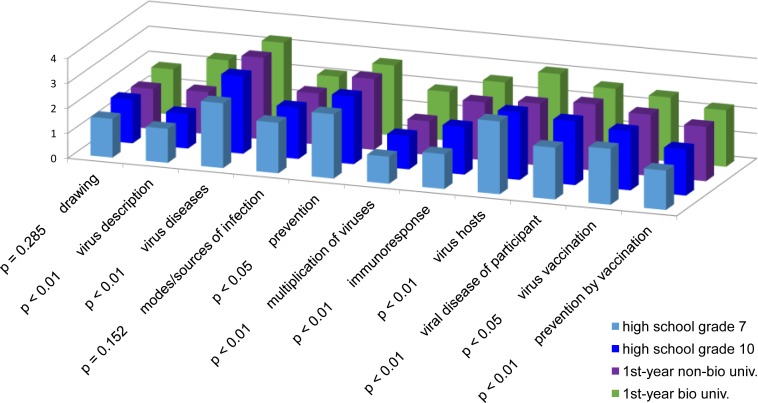
Average knowledge levels for each group along with p-levels for the ANOVA group effects.

Descriptive statistics of total virus-related knowledge for each of the four groups are given in Table 2. The ANOVA revealed a highly significant group effect for total knowledge (*F*(3, 642) = 44.17, *p* < 0.01, η^2^p = 0.17). Specific post-hoc tests by means of the Tukey test showed significant differences between all groups (*p* < .01) with the exception of 1^st^ year non-biology students and grade 10 high school students, which were only at a trend level (*p* = 0.05). Comparing the knowledge levels between the four groups, several interesting facts emerged: Biology students reached the highest overall knowledge levels, with non-biology students coming second, followed by grade ten students and finally by grade seven students (Table 2). With respect to specific knowledge categories, biology students almost always scored highest. This was true particularly for the description of a virus, its multiplication, and keywords indicating how the immune system would react to a viral infection (Fig 1, S15 Table). Univariate ANOVAs performed separately for each item revealed that significant knowledge differences were apparent in almost all items of the questionnaire, except for “drawing” (*p* = 0.29) and “modes/sources of infection” (*p* = 0.15), where no significant group effects were found (Fig 1). In some categories, grade 10 students even surpassed or at least equaled non-biology university students, e.g. with drawings, infection modes, and virus multiplication (Fig 1, S15 Table).

**Table 2:** Total knowledge levels for each group.

	Min.	Max.	M	SD
High school grade 7	13	31	21.34	3.82
High school grade 10	12	35	23.63	4.24
1^st^ year non-bio univ.	14	37	24.81	4.58
1^st^ year bio univ.	18	43	27.51	5.29

**Fig 2:**
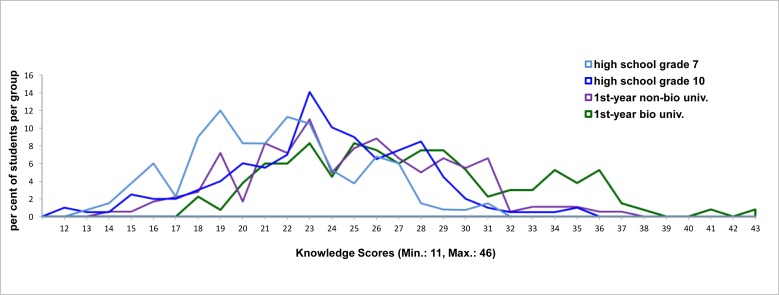
Individual knowledge levels reached by participants from the different groups.

**Fig 3:**
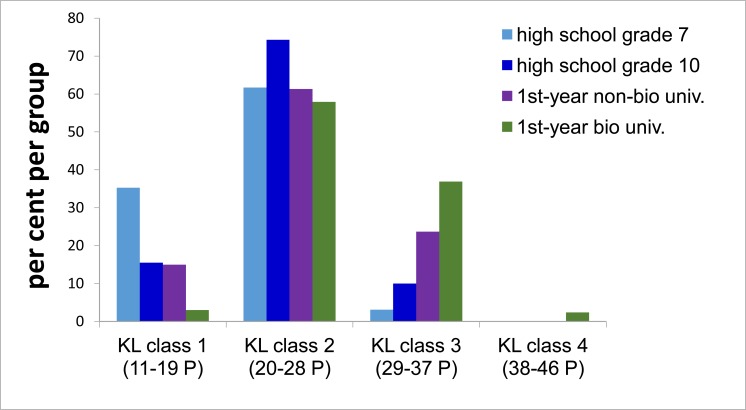
Distribution of students of each group among knowledge level (KL) class 1 (lowest) to 4 (highest).

The comparison of the four groups with respect to total knowledge also showed that the gap between grade 7 students and both grade 10 and non-biology first year university students, and between the latter two and biology first year university students was greater than between grade 10 and non-biology university students (Table 2, Fig 2). Furthermore, only three biology students, but none other, reached the highest total knowledge class (Fig 3).

### Correlations between items relating to specific virus knowledge

Correlations between items relating to specific knowledge about viruses were generally rather low (though mainly significant due to large sample size), and will thus not be discussed in more detail. There were, however, two notable exceptions: knowledge about prevention against specific viral diseases by vaccination was correlated with knowledge about names of viral diseases to a somewhat higher degree (*r* = .4; *p* < 0.01) and with knowledge that vaccination against (some) viral diseases is generally possible (*r* = .59; *p* < 0.01) (S16 Table).

### Correlations between knowledge and demographic variables

There were no significant correlations between virus related total knowledge and student’s sex and the number of their family members who work in a medical or biological field. However, analyses of potential associations between knowledge and demographic variables were complicated on the one hand by restricted variance (e.g., too few non-native students), and on the other hand by an unequal distribution of sexes particularly within both university groups (see Table 1).

### Association between knowledge and school

There were no significant differences with respect to knowledge as a function of school class, neither for grade 7 students (*F*(4, 128) = 0.65, *p* = .63), nor for grade 10 students (*F*(4, 194) = 1.48, *p* = .21).

### Students’ concepts on viral biology/health issues/Ebola

The following section provides quantitative and qualitative insights into the various concepts students provided concerning virus biology, health issues, and Ebola.

#### Brainstorming

The number of total concepts based on means (concepts per number of students in this group) was almost similar for university and grade 10 high school students (4.84/4.88/4.34 per biology/non-biology/grade 10 student). Grade 7 students provided much fewer concepts (3.32 per student).

University students studying biology offered much more concepts relating to the cellular infection processes during a virus infection (including virus multiplication) and to immunoresponse (0.72 concepts per biology university student vs. 0.32/0.2/0.03 per non-biology university/grade 10/grade 7 student). The same was true for virus biology in general (0.87 concepts per biology university student vs. 0.35/0.31/0.26 per non-biology university/grade 10/grade 7 student).

The number of misconceptions regarding viruses as some kind of living organism (e.g. a cell or bacterium) was similar for all groups (17.29%/12.15%/15.08%/16.54% of biology/non-biology/grade 10/grade 7 students), but much more biology students classified viruses as (potentially) non-living already during brainstorming: 24% vs. 3% (grade 7 and non-biology students) and 5% (grade 10 students).

High school students used moral traits such as “evil” more often than university students (8 and 5% for grade 10/7 vs. 1 and 3% for bio/non-bio students) and connected the term “virus” almost twice as often with computer programs. One grade 10 student explicitly wrote: “I think most viruses are mean”.

Concepts relating to medical topics such as diseases or treatment were similar in number for all groups.

No consistent pattern was found for concepts in connection with transmission and infection (S1 Table/Question 1).

#### Topics which required more information in the view of participants

The number of biology students who did not mention a single aspect they would see the need for more information was by far lower than that of any other group. Conversely, biology students showed the widest variety in aspects they would want to know more about, high school students the lowest (2.13 concepts per biology university student/ 1.53 per non-biology university student /0.87 per grade 10 student /1.19 per grade 7 student).

Biology students were more interested in biological (e.g. how viruses replicate, their biology/structure) and medical topics (e.g. diseases, treatment) than the other groups. For example, the number of concepts per participant relating to virus action and replication in the human body and host responses was 0.29 for biology students, 0.08 for non-biology students, 0.07 for grade 10 and 0.08 for grade 7 students.

Similar numbers from all groups were interested in the origin and evolution of viruses (10–22%) and, generally, in getting more information (4–8%) (S2 Table/Question 2).

#### Drawings

**Fig 4:**
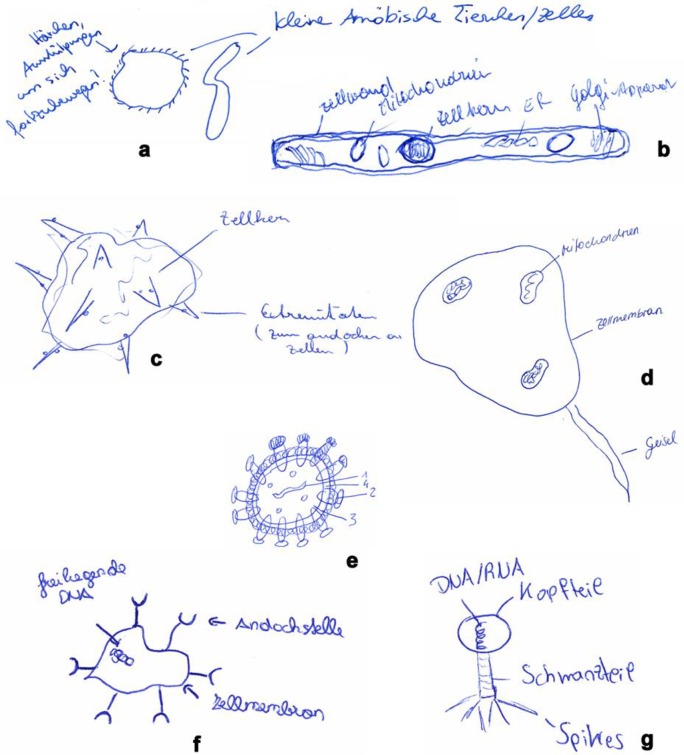
Examples for virus drawings from first-year biology students. The four drawings in the upper half represent pro-/eukaryotic cells, the three in the lower half drawings which come close to the true structure of a virus. a. labeling reads: “hair, protuberances to move forward” (left), “little amoeboid animals/cells” (right). b. labeling reads: “cell wall”, “mitochondria”, “nucleus”, “ER”, Golgi-apparatus”. c. labeling reads: “nucleus”, “extremities (to dock onto cells)”. d. labeling reads: “mitochondrion”, “cell membrane”, “flagellum”. e. labeling reads: 1 = RNA, 2 = tunnel proteins, 3 = matrix, 4 = membrane. f. labeling reads: “free DNA”, “docking place”, “cell membrane”. g. labeling reads: “DNA/RNA”, “head”, “tail”, “spikes”. Descriptions these students provided for a virus: a. “A disease-causing organism.” b. “A unicellular organism which is specialized to smuggle itself into the body and which transmits diseases.” c. “An alien cell with negative effect on immune system of body.” [This student also noted in the brainstorming, that “viruses are cells, which negatively influence or even kill other cells.”] d. “Very tiny organism.” e. “An organism, which integrates its RNA into the DNA of other cells and then kills this cell.” f. “Cell different to body cells, which weakens the immune system of the human or animal body.” [This student noted in the brainstorming, that viruses are “no living beings.”] g. “No living being; needs host to multiply. Very small. Antibiotics often without effect.”

69.9% of biology, 61.9% of non-biology, 81.4% of grade 10, and 90.2% of grade 7 students delivered a virus drawing. Drawings consisted of a wide range from simple dots or circles (many grade 7 students) to intricate drawings which were either (partially) correct or totally wrong. Fig 4 gives examples for drawings from first year biology students. As can be seen when comparing drawing and virus description (see figure legend), the drawing often provides a good indication of the lack of understanding a student has about the nature of a virus.

#### Virus description

While there was little difference between groups in relative number of students who left this item blank (13.53% - 18.23%), biology students outnumbered by far all other groups with respect to biological concepts such as virus action at/in host cells or virus structure/biology. For example, 32% of biology students related to virus structure/biology, but only 19% of non-biology students, 9% of grade 10 and 4% of grade 7 students. It is worth noting that several biology students classified viruses as some kind of organism; however, this might be explained by the frequent use of the term “microorganism” for viruses in textbooks and on the internet. Viruses were regarded as something (potentially) non-living (e.g. a particle) by 11% of biology students as opposed to only 4% from non-biology and grade 7 students and 3% from grade 10 students.

Few differences between university and high school students were found for concepts with respect to transmission/infection, diseases, and viruses as some kind of carrier of diseases.

Biology students had the highest number of different concepts used for virus description (1. 86 per student) with non-biology students showing only slightly more concepts than school children (1. 48 vs. 1.21/1.31 per grade 10/7 student) (S3 Table/Question 5).

#### Viral diseases

University students named more viral diseases, but also slightly more wrong diseases than younger students. This was mainly due to the frequent naming of malaria/*Plasmodium*, which younger students often simply do not know about. This disease is dealt with intensively in higher grades in many Austrian schools. Several students connected this complex disease with a virus (10% of biology, 6% of non-biology/grade 10, and 1% of grade 7 students).

HIV, Ebola, and influenza were by far the most often referred to viral diseases in all groups. HPV/cervical cancer was named more often by female than by male participants, even though its occurrence was generally scarce (biology students: 3 female/0 male; social science students: 5/2; grade 10: 5/1, grade 7: 0/1).

Students from grade 7 named cancer much more often than any other group (9% vs. 0–1% in other groups).

A relatively high number of students from all groups named bacterial diseases (6–15%) (S4 Table/Question 6).

#### Sources for infection

Few differences were observed between the four groups for possible ways of catching a viral disease. Biology students offered slightly more concepts (1.8 per student vs. 1.52–1.65 in the other groups) (S5 Table/Question 7).

#### Modes of prevention

In general, few differences between groups were found for ways to prevent becoming infected with a virus. However, university students in general noted more possibilities than school children, with younger children naming fewer concepts than older ones (2.08 and 2.07 concepts per biology/non-biology student, 1.86 and 1.47 per grade 10/7 student). All groups referred to cautious behavior when dealing with ill people, including protective clothing, with comparable frequency. Hygiene was placed top amongst all groups, with hand washing as the most prominent factor. Yet there were some age dependent differences: Use of disinfectants (0.11/0.18/0.07/0.02 concepts per biology/non-biology/ grade 10/grade 7 student) and contraception (0.22/0.18/0.17/0.11 concepts per biology/non-biology/ grade 10/grade 7 student) were more often named amongst older participants.

Relatively few participants named vaccination to prevent viral diseases, with biology students referring to this concept more than twice as much than any other group (29% vs. 12%/14%/8% for non-biology/grade 10/grade 7 students).

University students referred to various means of strengthening the immune system much more often than younger participants, particularly to diet and sports (18% and 25% (biology/non-biology students) vs. 10% and 9% (grade 10/grade 7 students)) (S6 Table/Question 8).

#### Multiplication of viruses

For this topic, up to two thirds of all non-biology and high school students did not provide any entry while this figure was much lower for biology students (one third). Also, biology students listed by far the most concepts. Unsurprisingly, the majority of grade 7 students did not provide answers here, as they had not encountered this topic within their curriculum yet.

Similarly, biology students outnumbered the other groups in almost all categories by far and generally exhibited more biological knowledge. This is exemplarily shown for some categories: Concepts relating to injection of viral DNA/RNA, to an alteration of the DNA of the host cell, or to the destruction of the host cell were found about five times more often amongst biology students than amongst participants of other groups. On the other hand, misconceptions such as virus multiplication via self-replication were found in similar proportions for university and grade 10 high school students.

The following concepts were most prominent: viruses attack cells; the infected cell produces new viruses; viruses multiply by host cell division. This indicates that participants did have some correct knowledge about host-virus interactions. However, the fact that even amongst university students up to two thirds of participants did not provide any answer, and that several answers were wrong (e.g. virus replication by own division) points to the need for more intense and detailed education concerning virus biology. Furthermore, apart from some biology students there were only few references to genetic material of a virus entering the host cell and (mostly) its DNA (S7 Table/Question 9).

#### Response of the immune system

As with the previous category, far more biology students left an entry here compared to other participants (91%/80%/73%/58% of biology/non-biology/grade 10/grade 7 students). However, differences in variety and frequency of occurrence of concepts were less pronounced. For example, antibodies were mentioned in similar proportions by biology, non-biology and grade 10 students, with the exception of grade 7 students, most likely because the latter had not yet been taught this topic at school.

However, university students related to specific host defense cells (e.g. T-cells, macrophages) much more often than school children (41% and 28% of biology/non-biology students vs. 11% and 17% of grade10/grade 7 students), which again is very probably due to the fact, that these aspects are usually dealt with in more detail in higher classes.

A large proportion of members from all groups mentioned symptoms, especially elevated temperature, as means to get rid of viruses (14–22%) (S8 Table/Question 10).

#### Viral diseases prevented by vaccination

Overall, about two thirds of university students, but less than half of school children, provided an answer to this item. This was reflected in concept variety, which was much higher amongst the former (1.1 and 0.98 concepts per biology/non-biology student vs. 0.62 and 0.51 concepts per grade 10/grade7 student).

In parallel, there was a gradient across age for correctly named viral diseases against which vaccination can offer (some) protection: Biology students named the most (1 disease per student), grade 7 students the least number of correct diseases (0.37 diseases per student). Conversely, fewer biology students (2%) than participants from other groups (6–7%) named bacterial diseases.

As with the list of viral diseases in general, HPV was named here more often by female than by male participants: 8 out of 9 biology students, 6 out of 6 non-biology students, and 7 out of 9 grade ten students naming HPV to be prevented from by vaccination were female. This shows that awareness about vaccination against this disease is scarce and, if at all, mostly present among female students (S9 Table/Question 21).

### Self-assessment concerning school as a source of general knowledge about viruses

**Fig 5:**
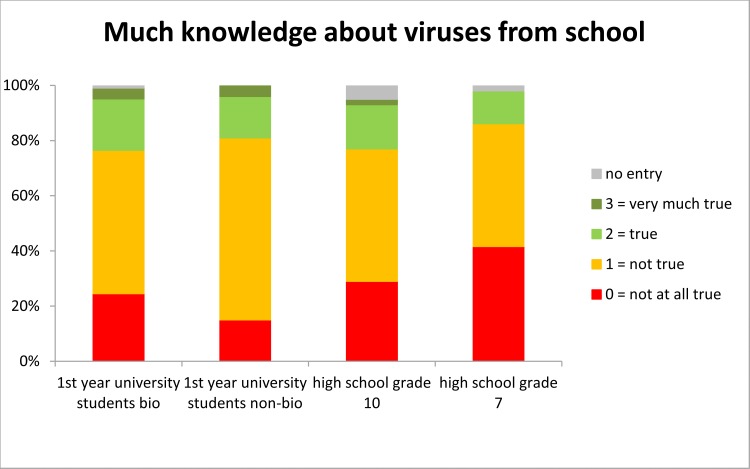
Self-report of amount of virus-related knowledge gained from school.

Across all groups less than a quarter of participants believed that they had gained much or very much knowledge about viruses at school (Fig 5). On the other hand, virus related knowledge was acquired from other sources than school according to self-report by only less than 20% of participants with one notable exception: Almost 40% of grade 7 students reported to have been informed about viruses outside school (S17 Table/Question 12), which might partly refer to information concerning Ebola in the media. Furthermore, the vast majority of participants felt that their teachers should equip them with more knowledge about viruses with almost 50% of biology students making this very urgent in comparison to only a third of non-biology university and a quarter of all high school students (S18 Table/Question 20).

### Desire for more virus-related knowledge

**Fig 6:**
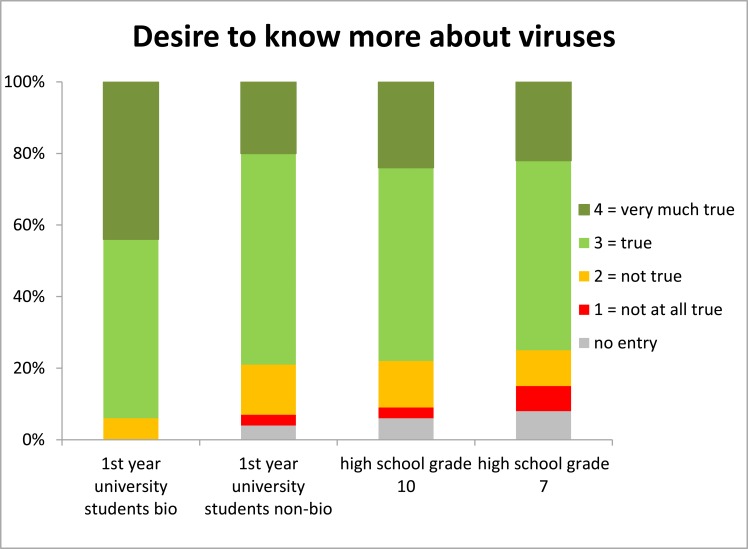
Self-report of interest in learning more about viruses.

There was little difference between non-biology university and high school students concerning their interest in virus-related information. However, biology students formed the by far most interested group. In all groups more than 75% expressed their desire to learn more about viruses (Fig 6).

### Knowledge about own virus infection in the past

This item was designed to test whether participants were aware of the fact that some diseases they very likely had experienced (e.g. influenza, chicken pox) had had a viral cause. Our data show that adolescents and young adults were mostly well informed in this respect. However, more than one third (37%) of grade 7 high school students were unsure whether they had had a viral disease (as opposed to 12–16% of participants from the other groups) (S19 Table/Question 18).

### Ebola-related knowledge

In this paper, we will only superficially deal with answers referring to Ebola related knowledge, since this will be a topic of a different publication. However, some aspects deserve to be mentioned here, because they relate to the function of school and media as sources for information about this virus.

#### Probable source of first Ebola infection of current outbreak

Our data show that information of participants was generally correct in that the two most often named causes were animals (with some students even referring to bats) and contaminated food. A few participants related to burial rites, which, although wrong, proves a close following of media coverage since transmission of the Ebola virus often occurred because of insufficient protection during burying the dead (S10 Table/Question 16).

#### Location of the current Ebola epidemic

As with the previous item, the answering patterns here were similar for all groups, even for wrongly named countries/regions. However, university and grade 10 students more often precisely named affected countries or, at least, West Africa as the most affected region compared to grade 7 students (“West Africa”: 19.55%/21%/16.58%/5.26% biology university/non-biology university/grade 10/grade 7 students) (S11 Table/Question 15).

#### Source for knowledge about Ebola

A very high proportion of all groups reported to have gained Ebola-related knowledge from classic media. In modern times it is interesting to note that 50% and more from all groups seemed to have read relevant newspaper articles and not only relied on television or the internet. Biology students named the internet as a source for information by far less often than all other participants (2% vs. 30%/26%/13% of non-biology/grade 10/grade 7 students).

School as a source for information concerning Ebola was generally rarely named, but more often for school children than for university students (7%/3%/ 12%/ 14% for biology university/non-biology university/grade 10/grade 7 students). The same was true for family (5%/2%/11%/ 11%). Probably, this mirrors the time of occurrence of this disease, when several university students had already left school. However, it is still interesting to note, that even school children apparently rarely heard about Ebola at school (S20 Table/Question 14).

#### Self-assessment of Ebola-related knowledge

Almost all participants from the three older age groups declared to be well informed about Ebola, while almost two third of grade 7 students believed contrarily (S21 Table/Question 13).

## Discussion

Our study shows that knowledge concerning viruses, their biology and their importance as causal agents for various diseases distinct from bacterial diseases is rather fragmentary even among university students, although it should be remarked positively that knowledge did increase with age and, thus, students seemed to have learnt aspects of viral biology at school, which confirms our hypothesis. As expected, first-year biology university students were performing better than all other groups concerning health issues and especially aspects of virus biology and structure, even though they had not met these subjects at their university courses yet. Since this group was also by far the most interested with respect to viruses, it may be deduced that interest and retaining virus-related knowledge from school and/or acquiring further knowledge from other sources out of intrinsic motivation might have contributed to the better performance of this group. Furthermore, it was surprising to find that grade 10 students surpassed first-year non-biology university students in some knowledge categories concerning virus biology. Possibly, this was due to more recently acquired relevant knowledge at school.

It should also be remarked here, that our scoring system does not differentiate between the kind of correct and incorrect answer(s) per item. Instead, it compares relative levels of knowledge, since for all open-response items, the same item-specific knowledge level could be achieved by providing different keywords.

Of course, knowledge is distributed in different breadth and depth in the various grades at school. Consequently, grade seven students, for example, will not be able to answer questions concerning multiplication of viruses, simply because they did not learn this at school yet. Table 3 shows which of the eleven items used for assessment of virus-related knowledge could have been answered by the respective group according to demands from the Austrian curriculum and a survey of popular Austrian biology text books used for high school.

**Table 3:** Knowledge students of the various age groups could have had according to official school curricula and school text book analysis.

Item	Grade 7^1^	Grade 10	University students^2^
Virus drawing		x	x
Virus description		x	x
Viral diseases	x	x	x
Modes of /sources for infection with a virus	x	x	x
Prevention against infection with a virus	x	x	x
Multiplication of viruses		x	x
Response of immune system to virus infection		x	x
Potential hosts for viruses	x	x	x
Former viral disease of participant (self-report)	x	x	x
Accepting the possibility of protection against viral diseases by vaccination	x	x	x
Naming of viral diseases which may be prevented by prior vaccination	x	x	x

^1^ Virus-related topics in grade 6 usually only concern health issues.

^2^ In grade 11 or 12 (depending on school type) students may learn virology in more depth; in particular, if the school offers electives in biology, which very often have a special focus on medical topics, since many students participating in these elective courses plan to study medicine.

Yet the fact that even some university students studying biology had serious misconceptions concerning virus biology and related health issues should be of strong concern for teachers, health educators and physicians. For example, one 19-year old Austrian biology student mentioned “antibiotics” and described a virus as a “bacterium”. This central misconception was prevalent to a relatively large degree in all groups—and may directly lead to a wrong understanding of treatment. Further work is necessary to understand why bacterial diseases have been understood as being caused by a virus, or if this was due to the fact, that these students could not differentiate between the two.

Furthermore, the observation that non-biology students and grade ten students did not differ significantly with respect to their overall knowledge clearly points to the need for intensifying and, possibly, modifying teaching this topic, too. This is reflected in participants’ notion that their knowledge about viruses acquired at school is far from satisfactory. Also, virus-related knowledge of university students not enrolled in medical/biology courses will very likely not improve–unlike of those studying biology–and therefore mirrors the knowledge prevalent amongst those members of our society which have passed their final high school exams. People with lower education will most likely know even less about viruses, which requires further studies.

Group similarities in knowledge concerning modes/sources of infection may reflect knowledge gained at school and possibly at home: Health education usually puts much emphasis on how a disease might be caught. Experience from home and from school might also explain the low difference in concept variety between groups concerning prevention against viral diseases. With respect to the repeatedly given concept “contraception” as a way to protect oneself against sexually transmitted diseases it should be noted that contraception *per se* does not offer such protection. Even though many participants naming this concept might have had condoms in mind (which may also not always offer total protection), it cannot be ruled out that other ways of contraception had misleadingly been thought of. This becomes clear in the case of one 7^th^ grade student, who explicitly referred to the birth control pill. Here, a more detailed sexual education seems vital in preventing such potentially dangerous misconceptions. Concerning healthy living as a way to prevent a viral disease, we found a strong sex bias for high school students (data from university students could not be used here, because of the unequal distribution of sexes among them): Female participants referred much more often to dietary aspects than males: 5 out of 7 10^th^ grade students naming this concept were female, as were 8 out of 9 7^th^ grade students. This indicates a higher awareness concerning healthy living for female students. Yet even though a generally healthy living may help to decrease the risk of becoming ill, there is no consistent proof in medical literature so far that such behavior indeed diminishes the likelihood of viral infections when being confronted with a defined viral load.

Visual aspects of a virus (e.g. using electron microscopy pictures) are apparently discussed in lesser detail at school, even in grade 12, since no significant differences were found between groups. However, a closer look revealed that there were differences between groups when only those students were regarded who had drawn a virus more or less correctly: While first-year biology students relatively often drew a bacteriophage (a virus infecting and replicating within a bacterium), all other groups provided almost exclusively sketches from filiform (thread-like; as Ebola) or retroviruses (e.g. HIV), which may reflect influence of either the media or the school, since pictures of the Ebola virus were regularly printed in the papers at the time of questionnaire administration, and HIV is a compulsory subject at school (Table 4).

**Table 4:** Partially or totally correct virus drawings of students according to virus classification.

Virus type	Grade 7	Grade 10	1^st^ year univ. non-bio	1^st^ year univ. bio
Filiform	18	18	8	4
Retrovirus	13	33	24	17
Adenovirus	3	1	0	2
Bacteriophage	0	1	7	17

It is interesting to note that correlations between the various knowledge items were generally low with the exception of knowledge about prevention against specific viral diseases by vaccination, which was correlated with knowledge about names of viral diseases and with knowledge that vaccination against (some) viral diseases is generally possible. Thus, several students did apparently not only know various viral diseases but also against which of those one may be vaccinated. This may be due to personal experience (e.g. vaccination against tick-borne encephalitis, which is regularly offered in Austrian schools).

Another interesting fact was that we found no significant difference between students from the same grades but different schools among the tested seventh and tenth graders. This indicates that virus-related information had been delivered rather equally in the five high schools which participated in this study. Results might have been different for other school types, but this remains to be studied in future work.

Wrong or non-existent knowledge about viruses is not only of academic interest. For example, misconceptions with respect to transmission and prevention of HIV have been found for a substantial proportion of high school students in Ethiopia [14], India [15], Turkey [16], Iraq [2], Nigeria [3], South Africa [17], the US [5], for African American college students [18], and German, British and Russian nursing students [1,19,20]. Similar results were found for nursing or medical students concerning HPV in Scotland [21], Nigeria [22] and Switzerland [23], and for high school students with respect to influenza in France [4] and the US [12].

An exemplary case is HIV, since it has been extensively covered both in the media (news and movies) and at school in many countries, and is still one of the most life-threatening viral diseases. Teaching virus biology and methods to prevent infection may thus have direct effects on students’ health. For example, the level of knowledge was found to correlate with the perceived risk of contracting sexually transmitted diseases amongst adolescents in Laos [24]. This may also be true concerning the demands of many patients to be unnecessarily treated with antibiotics when suffering from a viral disease, which is not only without effect, but may additionally result in an increasing number of resistant bacterial strains.

While demographic factors seemed to have had no influence on virus related knowledge in our study, the role of the media should not be underestimated: For many high school students, media and especially the internet are apparently an important source of information [3,14,16]. This requires further attention, since such information may not always be correct and comprehensive knowledge about diseases should be delivered in health education at school.

Of course, the analysis of knowledge gaps can only be the first step towards the development and implementation of school material and teaching units, which help to long-lastingly improve virus related knowledge. In particular, virus structure and biology seems to have been seriously neglected at school.

How urgent this is becomes clear from the recent European Commission’s Eurobarometer on Antimicrobial Resistance: 46% of participants from all over Europe falsely stated that antibiotics kill viruses, 11% could not answer the question. Interestingly, correctness of answers differed widely between countries. In Sweden, only 22%, but 38% of the British, 45% of the German, and 63% of the Austrian study participants delivered the wrong answer. Overall, far more than half of Europe’s population did not know how to correctly handle antibiotics, and that this medication is not efficient in treating viral diseases. More specifically, 36% of Europeans believed that antibiotics are helpful in treating colds and flu (the two most commonly experienced viral diseases), 8% were uncertain [25]. Teaching virus biology, structure and treatment at school seems thus a very important issue. As we have shown recently for epilepsy such units can be very effective [26]. It should be encouraging to teachers and health educators, that the interest in virus-related topics was generally very high amongst participants from all groups.

## Limitations

The work presented here faces some limitations. First, the high-school student population was not randomly chosen, but based on school’s willingness to participate. This, however, was unavoidable since students cannot participate without teacher and parental consent. Secondly, these students all attended high schools. Further studies are needed to test middle-school students’ knowledge about viruses. Third, the same applies to the adult population tested here: subsequent work should include same-age groups of different professions, not just university students.

Concerning item phrasing, some adjustments for future studies may help to extract even more knowledge: Participants should be explicitly asked to provide as many correct answers as they could think of (e.g. for viral diseases) to avoid that some students may have thought that naming a few might be sufficient.

The methodological approach has two drawbacks: It does not allow for in-depth tracking of origins for specific concepts as interviews do, and it is not as easily rated as multiple-choice items. However, the comprehensive open-response questionnaire including the drawing task seems a feasible alternative to simple multiple-choice questionnaires on the one and interviews on the other hand. While the first are easy to handle, they may be limited in their access to participants’ actively accessible knowledge and understanding used for decision making [27,28], all the more since the correct answer is already provided in multiple-choice items. The analysis of interviews, though, is very time-consuming and thus restricted to a limited number of students. Questionnaires of the type used here allow a much more detailed reconstruction of students’ knowledge and understanding than multiple-choice questionnaires while still making use of a large number of participants without the need to record and transcribe interviews. Furthermore, since both the pilot and the main study showed that students could handle all items well, this instrument can be recommended for use for all secondary students and adults, which simplifies further testing for virus knowledge.

## Supporting information

S1 Table Categorization of answers for brainstorming. (XLS)

S2 Table Categorization of topics for which participants required more information. (XLS)

S3 Table Categorization of virus descriptions. (XLS)

S4 Table Categorization of named viral diseases. (XLS)

S5 Table Categorization of named sources for infection. (XLS)

S6 Table Categorization of named methods for prevention. (XLS)

S7 Table Categorization of description of virus multiplication. (XLS)

S8 Table Categorization of description of immunoresponse. (XLS)

S9 Table Categorization of named diseases against which prevention by vaccination is possible. (XLS)

S10 Table Categorization of named origin of Ebola epidemics. (XLS)

S11 Table Categorization of named locations of Ebola occurrence. (XLS)

S12 Table Rating scheme for assessing virus-related knowledge. (DOCX)

S13 Table Categories for coding virus drawings. (DOCX)

S14 Table Individual data and ratings. (XLS)

S15 Table Item-wise knowledge levels per group. (XLS)

S16 Table Non-parametric correlations (Spearman-Rho) between knowledge items. (XLS)

S17 Table Non-school knowledge of students about viruses (self-report). (XLS)

S18 Table Sufficiency of virus-related school-knowledge (self-report). (XLS)

S19 Table Knowledge about own viral diseases in the past (self-report). (XLS)

S20 Table Sources for knowledge about Ebola (self-report). (XLS)

S21 Table Knowledge about Ebola (self-report). (XLS)

S1 Questionnaire Questionnaire for high school students. (DOCX)
