# Supplementary material for: "The evil virus cell": Students‘ knowledge and beliefs about viruses
Source: PLoS One. 2017 Mar 28;12(3):e0174402. doi: 10.1371/journal.pone.0174402 (PMC5370109; doi:10.1371/journal.pone.0174402)
Supplement: S12 Table — (DOCX) [file pone.0174402.s012.docx]

**S12 Table. Rating scheme for assessing virus-related knowledge.**

| 1. **Virus drawing** | **Knowledge level** |
| --- | --- |
| no drawing/wrong drawing | 1 |
| correct, but very simple drawing (e.g. filiform for ebola-virus without further details) | 2 |
| partially correct drawing showing detailed virus organization | 3 |
| correct drawing showing virus organization | 5 |

| 1. **Virus description** | **Knowledge level** |
| --- | --- |
| no/wrong/unspecific answer (e.g. virus = "cell", "micro-organism", "disease", "disease carrier", "substance", "no nucleus/mitochondria" [which is partly true also for bacteria]) | 1 |
| virus ≠ "bacteria"; "unclear whether living organism", "antibiotics non-efficient" | 3 |
| virus ≠ "cell", "no metabolism", "particle", "non-living"; etc.; also, if strong indication that virus regarded as non-cellular | 5 |
|  |  |
| *Further rules:* |  |
| 1. *if further answers, e.g. from brainstorming and drawing, indicate that students are well aware that viruses are non-living, than "micro-organism"/"living organism" as answer is not counted, since many school texts/internet pages classify viruses as micro-organisms* |  |
| 1. *if further answers, e.g. from brainstorming or from virus description, are severely wrong (e.g. "multiplies outside cell", "self-multiplication"; "toxin", "antibiotics"), than reduction by one level, e.g. from 3 to 2* |  |

| 1. **Viral diseases** | **Knowledge level** |
| --- | --- |
| 0 correct diseases | 1 |
| 1 correct disease | 2 |
| 2 correct diseases | 3 |
| > 2 correct diseases | 4 |
|  |  |
| *Further rules:* |  |
| 1. *for each non-viral disease subtract 1 correct diseases to account for the fact that some participants seemed to have listed various diseases without differentiation between viral and non-viral diseases* |  |
| 1. *diseases with might have other origins, but are predominantly viral - such as bronchitis or gastroenteritis, are counted as correct* |  |
| 1. *irrelevant terms such as "temperature" (symptom) or "droplet infection" (modes of infection) are not counted* |  |
| 1. *AIDS / HIV treated synonymously* |  |
| 1. *"cancer" not counted, because too unspecific* |  |

| 1. **Modes of /sources for infection with a virus** | **Knowledge level** |
| --- | --- |
| no/wrong answer | 1 |
| 1-2 correct answers | 2 |
| > 2 correct answers | 3 |
|  |  |
| *Further rules:* |  |
| 1. *each wrong answer: -1* |  |
| 1. *"immunodeficiency", etc. regarded as wrong, since this refers to probability of disease outbreak in patient, not to contact with the virus* |  |
| 1. *"air" counted as correct, since very likely referring to droplet infection via coughing/breathing etc.* |  |

| 1. **Prevention against infection with a virus** | **Knowledge level** |
| --- | --- |
| no/wrong answer | 1 |
| correct but very general, leaving room for interpretation (e.g. "contraception", "education", "information", "prevention", "no drugs", "hygiene", "no travels to West-Africa", etc.) | 2 |
| 1-2 correct and specific methods (e.g. "condoms", "no exchange of body fluids", "hand washing", etc.) | 3 |
| > 2 correct and specific methods (see above) | 4 |
|  |  |
| *Further rules:* |  |
| 1. *each wrong answer: -1* |  |
| 1. *"medication" regarded as wrong, since this refers to treatment, not to prevention* |  |
| 1. *answers refering to strengthening the immune system - either generally or specifically through vitamines, sports, etc., are not counted since literature is inconclusive here* |  |
| 1. *"stay home" regarded as correct, because it may refer to preventing transmission of own virus to others* |  |

| 1. **Multiplication of viruses** | **Knowledge level** |
| --- | --- |
| no/wrong/unspecific answer (e.g. "conjugation", "eats host cells", "attacks body/cells", "proliferation in host/host cells", etc.) | 1 |
| 1 of the list below: | 2 |
| 2 of the list below | 3 |
| 3 of the list below | 4 |
| > 3 of the list below | 5 |
|  |  |
| ***List:*** |  |
| attachment to host cell/infection of cell |  |
| virus DNA/RNA injected into host cell |  |
| viral DNA integrated into host DNA |  |
| (host) cell division |  |
| misuse of host metabolism for viral DNA/RNA amplification |  |
| synthesis of protein coat |  |
| lysis of host cell |  |
| virus particles set free |  |
|  |  |
| *Further rules:* |  |
| 1. *each wrong answer: -1 (wrong answers weighed higher, because very often making apparent severe misconceptions; e.g. virus replication by self-multiplication, by eating of host cells, by conjugation, etc., indicating that viral replication has not been understood/is not known)* |  |
| 1. *unspecific answers (e.g. "attacks body/cells", "proliferation in host/host cells"): no subtraction* |  |
| 1. *"inserts RNA into host cell DNA" counted as correct, although transcription step into DNA missing, because indicating a relatively detailed understanding of virus replication* |  |

| 1. **Response of immune system to virus infection** | **Knowledge level** |
| --- | --- |
| no/wrong (e.g. "antivirus", "antigen", "erythrocytes", "excretion", etc.) / unspecific answer (e.g. "blood/defence cells", "enzymes", "defence system", "defence substances", etc.) | 1 |
| "immune system", "temperature", *“inflammation, “sweating”, “mucus”* | 2 |
| 1 of the list below | 3 |
| 2 of the list below | 4 |
| ≥3 of the list below | 5 |
|  |  |
| ***List****:* |  |
| *antibodies* |  |
| *leucocytes* |  |
| *T-cells/B-cells/memory cell* |  |
| *immunoglobulines* |  |
| *macrophages/phagocytosis* |  |
| *virus recognition/marking/identification of foreign DNA* |  |
| *host cell death/apoptosis* |  |
| *exocytosis* |  |
| *destruction of foreign DNA/destruction of virus* |  |
|  |  |
| *Further rules:* |  |
| 1. *each wrong/unrelated (e.g. "shivering/vomitus/disease" = consequence of immunoresponse) answer: -1* |  |
| 1. *unspecific answers, e.g. "enzymes", defence system", “blood cells“ etc. not counted* |  |
| 1. *"immune system" counted as correct, because indication for knowledge/understanding of more than simple "defence"; but due to lack of specificity weighed lower than terms on list* |  |
| 1. *"temperature" counted as correct, because reason may be immune cell (e.g. macrophage) contact with viruses and their subsequent interleukin-1 production causing a rise in body temperature; however, since students very likely did not know these precise mechanisms and probably regard "temperature" as a simple body reaction to infection, this answer is only classified as level 2; similar rating for “inflammation, “sweating”, “mucus”, since precise mechanisms very likely not known, instead possibly reference to symptoms only.* |  |
| *Example: student provides one correct answer from the list, one wrong answer, and "immune system" --> Level 2, because "1 of the list below" is neutralized by the wrong answer* |  |
| 1. **Potential hosts for viruses** | **Knowledge level** |
| 1 host type | 1 |
| 2 host types | 2 |
| 3 host types | 3 |
| 4 host types | 4 |
| 5 host types | 5 |

| 1. **Former viral disease of participant (self-report)** | **Knowledge level** |
| --- | --- |
| no/uncertain | 1 |
| yes | 3 |

| 1. **Accepting the possibility of protection against viral diseases by vaccination** | **Knowledge level** |
| --- | --- |
| no/uncertain | 1 |
| yes | 3 |

| 1. **Naming of viral diseases which may be prevented by prior vaccination** | **Knowledge level** |
| --- | --- |
| no/wrong answer | 1 |
| 1-2 correct diseases | 3 |
| 3-4 correct diseases | 4 |
|  |  |
| *Further rules:* |  |
| 1. *each non-viral disease or each viral disease with no vaccination against: -1* |  |
| 1. *unspecific answers not counted* |  |
| 1. *hepatitis C vaccine is presently tested but not yet available, thus wrong* |  |
| 1. *"ticks" not counted, because could refer to viral meningitis (correct) or lyme disease (wrong)* |  |
| 1. *“HIV" vaccines are presently tested but not yet available, thus wrong* |  |
| 1. *"cancer" not counted, because too unspecific* |  |

**Additional rules:**

1. **Answers from brainstorming are to be considered for relevant items --> particularly concepts relating to virus biology/structure (e.g. "living"/"antibiotics" vs. "non-living"/"particle" for item *virus description*), to virus replication, to immune system response, and to viral diseases.**
2. **Answers from brainstorming are not to be considered, if not unequivocally interpretable (e.g. "saliva" or "blood" may relate to sources of infection, spread in body, or locations of virus in body; "bacterium" may refer to other diseases or hosts for bacteriophages, etc.).**
3. **In case of conflict (incorrect concept for same topic for one item, but correct concept for other item; including drawing) 🡪 no consideration of this concept.**

***Examples:***

1. **virus described as "non-living" in brainstorming, but as "cell" in item virus description); exception: concept refers to sth. else (e.g. cell as host)**
2. **virus described as different to bacteria (correct), but antibiotics as method for treatment or prevention (wrong)**
3. **"Antibiotics" in brainstorming counted as wrong when mentioned on its own, but not counted when student described a virus as "non-living"/"particle"/ “≠ bacteria" etc.), because it may then refer to the topic of how or how not a virus may be healed (e.g. as discussed in the media), not to the student’s belief that this is effective virus treatment (e.g. biology student NAPEHE).**
4. **If "bacterium" occurs in brainstorming, but other descriptions show that student DOES NOT regard viruses as bacteria (e.g. biology student JACLMA) 🡪 answer counted as correct, because occasionally viruses are presented as bacteriophages in conjunction with bacteria at school.**
5. **For items relating to drawing, description, own viral diseases and vaccination correct answers are valued higher than according to a linear scale, because they show a profoundly greater understanding of the respective topics (a linear scale would underestimate the improvement in knowledge development for this item and student).**

**Further remarks:**

**Within this complex scoring system two rating rationales may need further explanation:**

1. **Wrong answers were penalized with knowledge level reduction, because we noticed that**

**some students apparently wrote as much as they could without reflecting much on correctness; example: some students wrote down many different disease names when asked to provide names of viral diseases, but did not distinguish between viral and non-viral diseases - this may reflect some knowledge concerning diseases, but not knowledge with respect to specifically VIRAL diseases. Students, who provided fewer but correct answers, should thus score better.**

1. **Participants who left an item blank were also given the baseline knowledge level to acknowledge that there are students, who would only give an answer if they are certain of its correctness. Such students are admittedly not to be distinguishable (for a specific item) from students who left an item blank because they truly did not know the answer. However, those "make-it-completely-right-or-leave-it"-students would be overly punished otherwise. Within our system, such students should perform better, because they may score enough at other items. This can be illustrated with an example from our data: Comparing seventh grade students SOSIGE and ELANMA (both with Austrian mother tongue and from the same class), SOSIGE provides fewer answers for some items, but those given are usually better than those from ELANMA. Examples: SOSIGE refers to the concepts "transmission", "disease/infection" and even "particle" already during brainstorming, where ELANMA only relates to "disease/infection" and "computer". When it comes to the description of a virus, "SOSIGE" notes "contagious" and "no treatment" (true for most viral diseases), while ELANMA only refers to "contagious" and the general term "disease". Concerning diseases, ELANMA names "coughing" (only symptoms), "cold" (mostly caused by virus), and "plague" (caused by a bacterium), while SOSIGE only but correctly names "ebola". Comparing answers referring to multiplication of viruses, ELANMA wrongly writes "continuous growth", while SOSIGE left this item blank - probably being too uncertain to answer. This trend is also recognizable for their drawings: SOSIGE does not draw any sketch, while ELANMA creates a drawing which shows attached circles. In summary, it seems that SOSIGE is more honest and reflective in her answering (which is also more scientific), but consequently leaves some items blank, which are filled out by her classmate ELANMA, who often seems to just note what comes to her mind, without reflecting (or knowing) much about the truth of her answer.**

**As a consequence, for some items students with no answer and students with wrong answers may earn the same knowledge level. However, as conceptual change theory, in particular the importance of the so-called cognitive conflict (e.g. between a personal, non-scientific and a scientific view of a phenomenon)^1^ and experience from the classroom show, incorrect knowledge is not always easily corrected and may thus more difficult to work with (as an instructor) than uncertainty or even lack of knowledge, since it is much easier to sustainably convince students who have no pre-conceptions of a scientifically correct view compared with students who have misconceptions due to daily life experiences and/or non-scientific views taken up from their surroundings such as friends and family.**

**Literature:**

1 Treagust DF, Duit R. Conceptual change: a discussion of theoretical,

methodological and practical challenges for science education. Cult Stud of Sci Educ. 2008;3:297-328.
