# Supplementary material for: "The evil virus cell": Students‘ knowledge and beliefs about viruses
Source: PLoS One. 2017 Mar 28;12(3):e0174402. doi: 10.1371/journal.pone.0174402 (PMC5370109; doi:10.1371/journal.pone.0174402)
Supplement: S13 Table — (DOCX) [file pone.0174402.s013.docx]

**S13 Table. Categories for coding virus drawings.**

**0 = no drawing**

**1 = circle/points**

more or less blank round shapes with no further details

**2 = attached/connected circles/points**

round shapes with some kind of connection

**3 = shape(s) other than circles / not clearly categorizable**

oval, square, star, undefined polygon, rod, animal, mixture between different structures, not recognizable (e.g. sun-like with uneven surface)

**4 = monster/grimly-looking smiley**

obviously personalized shape

**5 = eukaryotic cell-like**

organization of shape includes some (not necessarily all) typical eukaryotic cell parts, such as nucleus, ribosomes, mitochondria

**6 = paramecium-like**

oval/round shape with ciliae and nucleus-like structure within

**7 = bacterium-like**

organization of shape includes some (not necessarily all) typical prokaryotic cell parts, such as plasmid, flagellum, etc.

**8 = worm-like with head and/or tail**

clearly recognizable “body” parts like head, segments, etc.

**9 = plain worm/filiform (ebola-like)**

worm-like structure, possibly with “spikes” (glycoproteins)

**10 = retrovirus (e.g. HI-virus; often dealt with at school)**

structure with circle-like central part plus surface with high number of ‘spikes” (glycoproteins), ideally showing internal details such as RNA molecule; partly correct

**11 = retrovirus (e.g. HI-virus; often dealt with at school)**

structure with circle-like central part plus surface with high number of ‘spikes” (glycoproteins), ideally showing internal details such as RNA molecule; totally correct

**12= adenovirus**

same as 10/11, but central structure icosahedral, partly correct

**13 = adenovirus**

same as 10/11, but central structure icosahedral, totally correct

**14 = bacteriophage**

partly correct

**15 = bacteriophage**

totally correct

**Additional rules:**

🡪 “Partly correct” refers to those drawing which obviously belong to a virus category, but important details are missing or wrong; e.g. only capsule for bacteriophage without neck and spikes, or wrong nucleic acid labeling (e.g. “DNA/RNA” instead of only the correct one).

🡪 If more than one drawing is present and these drawings are thematically separate (bacteriophage + bacterium counts as one) and one correct, one not, than max. level three, but category for the correct one noted.

🡪 Labeling to be included in categorization.
