# Supplementary material for: "The evil virus cell": Students‘ knowledge and beliefs about viruses
Source: PLoS One. 2017 Mar 28;12(3):e0174402. doi: 10.1371/journal.pone.0174402 (PMC5370109; doi:10.1371/journal.pone.0174402)
Supplement: S1 Questionnaire — (DOCX) [file pone.0174402.s022.docx]

***Dear Participant!***

***Thank you very much for filling out this questionnaire. It is very important for us to learn what you know about the topic “virus“ to be able to develop suitable school material. Therefore I would ask you to fill out this questionnaire thoroughly and honestly. Thereby you will contribute to improve teaching.***

***Many thanks!!!***

***Dr. Uwe Simon, Center for Biology Teacher Education, Graz University***

Code: first two letters of your surname

+ first two letters of your mother’s surname

+ first two letters of your father’s surname

(example: student‘s name **AL**OIS, mother‘s name **SO**NJA, father’s name **HU**BERT

🡪 **ALSOHU**)

Code: _____________

age: _____________

□ female □ male

mother tongue: ________________

nationality: __________________

number of family members who work in a medical or biological field (e.g. parents / aunts/uncles as nurses, physicians, biologists, etc.): ____________________

**Part 1**

1. ***Please note what comes to your mind when you think of the word “virus“ (keywords).***
2. ***Please note aspects of the topic “virus“ you would like to know more about.***

**Part 2**

**(From now on the word “virus“ refers only to biological viruses, NOT to computer software!)**

1. ***Draw a virus.***
2. ***Label your drawing.***

**Part 3**

1. ***Describe, what a virus is.***
2. ***Which viral diseases do you know?***
3. ***Give keywords, how one may catch a viral disease.***
4. ***Give keywords, how one may protect him-/herself against such an infection.***
5. ***Describe, how a virus proliferates in the human body.***
6. ***Describe, how the body defends itself against viruses which have entered the body.***
7. ***I have learnt a lot about viruses at school:***

*very much true □*

*true □*

*not true □*

*not at all true □*

1. ***I know a lot about viruses, but not from school.***

*□ yes □ no*

1. ***Do you have knowledge about the “Ebola virus“?***

*□ yes □ no*

1. ***If “yes”: from which source(s)?***
2. ***Where are currently many people suffering from Ebola?***
3. ***How has the first Ebola patient probably caught the disease?***
4. ***Are viruses dangerous for humans only, or can they also infect other organisms? Please tick off (you may tick off more than one answer):***

*humans □*

*animals □*

*plants □*

*fungi □*

*bacteria □*

1. ***I have had a viral disease at least once in my life:***

*□ yes □ no □ don’t know*

1. ***September 30th, 2014: The first case of Ebola was made public in the USA – an airplane passenger, who had probably become infected in an Ebola-struck country. Take the role of the disease supervisor of the Austrian government. Suddenly, a passenger collapses at Vienna airport and is brought to hospital with high temperature. The physicians diagnose Ebola. What needs to be done to prevent further spread of the virus (keywords)?***
2. ***I think, one should learn more about viruses at school:***

*very much true □*

*true □*

*not true □*

*not at all true □*

1. ***Do viral diseases exist against which one may be protected by a vaccine?***

*□ yes □ no □ don’t know*

1. ***If “yes”: which ones?***
2. ***If “no”: Why do you think there are no vaccines?***
3. ***I would like to know more about viruses:***

*very much true □*

*true □*

*not true □*

*not at all true □*

***Once again: Many thanks for your participation!!! ☺***
